# Using blockchain to log genome dataset access: efficient storage and query

**DOI:** 10.1186/s12920-020-0716-z

**Published:** 2020-07-21

**Authors:** Gamze Gürsoy, Robert Bjornson, Molly E. Green, Mark Gerstein

**Affiliations:** 1 Program in Computational Biology and Bioinformatics, Yale University, New Haven, CT, 06520 USA; 2 Department of Molecular Biochemistry & Biophysics, Yale University, New Haven, CT, 06520 USA; 3 Yale Center for Research Computing, New Haven, CT, 06520 USA; 4 Department of Computer Science, Yale University, New Haven, CT, 06520 USA

**Keywords:** Blockchain, Secure storage, Genomic data access log

## Abstract

**Background:**

Genomic variants are considered sensitive information, revealing potentially private facts about individuals. Therefore, it is important to control access to such data. A key aspect of controlled access is secure storage and efficient query of access logs, for potential misuse. However, there are challenges to securing logs, such as designing against the consequences of “single points of failure”. A potential approach to circumvent these challenges is blockchain technology, which is currently popular in cryptocurrency due to its properties of security, immutability, and decentralization. One of the tasks of the iDASH (Integrating Data for Analysis, Anonymization, and Sharing) Secure Genome Analysis Competition in 2018 was to develop time- and space-efficient blockchain-based ledgering solutions to log and query user activity accessing genomic datasets across multiple sites, using MultiChain.

**Methods:**

MultiChain is a specific blockchain platform that offers “data streams” embedded in the chain for rapid and secure data storage. We devised a storage protocol taking advantage of the keys in the MultiChain data streams and created a data frame from the chain allowing efficient query. Our solution to the iDASH competition was selected as the winner at a workshop held in San Diego, CA in October 2018. Although our solution worked well in the challenge, it has the drawback that it requires downloading all the data from the chain and keeping it locally in memory for fast query. To address this, we provide an alternate “bigmem” solution that uses indices rather than local storage for rapid queries.

**Results:**

We profiled the performance of both of our solutions using logs with 100,000 to 600,000 entries, both for querying the chain and inserting data into it. The challenge solution requires 12 seconds time and 120 Mb of memory for querying from 100,000 entries. The memory requirement increases linearly and reaches 470 MB for a chain with 600,000 entries. Although our alternate bigmem solution is slower and requires more memory (408 seconds and 250 MB, respectively, for 100,000 entries), the memory requirement increases at a slower rate and reaches only 360 MB for 600,000 entries.

**Conclusion:**

Overall, we demonstrate that genomic access log files can be stored and queried efficiently with blockchain. Beyond this, our protocol potentially could be applied to other types of health data such as electronic health records.

## Background

Genomic data collected from patients should be protected as it identifies the individual and is private information. With the decreasing cost of sequencing the amount and type of human-derived genomics and related data is increasing exponentially [1]. Private information leakages based on genomic data have been extensively studied for decades [2–7]; hence, sensitive genomic data is protected through controlled access, and access logs are stored for auditing potential malicious activities [8, 9]. Securely maintaining accurate usage logs of data access is imperative, because if usage logs are corrupted, changed, or lost, then auditing the access to extremely sensitive data is not possible.

Security and integrity of data can be achieved by focusing on two key requirements. The first is that the usage logs must not be lost, whether due to a software attack, a hardware or structural failure, or some other disaster. The second is that the usage logs cannot be manipulated. One method to ensure that usage logs are not lost involves storing multiple copies of the data, either within the same organization or across multiple parties. If the copies are stored at the same site, a structural failure could result in loss of the data; however, storing copies of data with multiple parties requires trust, and there is no guarantee that each copy of the log data is identical. Furthermore, even if data logs are stored within the same organization (perhaps across multiple sites), there is no guarantee that the logs have not been manipulated by an individual within the organization, whether by accident or intentionally. An ideal implementation would protect from both loss and manipulation. To guarantee that redundancies are identical and to protect against manipulation, we recommend moving away from relying on trusted parties or authority figures (individuals or organizations) and instead placing that trust in an algorithm. Such a tool could be employed by the implementation to provide clear outputs alerting users of changes in the logs. Blockchain technology employs an ideal implementation due to three key properties: decentralization, immutability, and security [10]. Decentralization prevents a single entity from controlling the data; immutability guarantees that past records cannot be altered; and security is ensured by protecting accounts with enhanced cryptographic methods [10].

A network blockchain, or chain, can be broadly thought of as a transparent, append-only list, which anyone within the chain network can see and append to with proper permission. Every node within the network locally stores a copy of the list, making it a decentralized system. When a node in the network appends an item to the list, an output ID is generated. This ID is dependent on properties of the appended item as well as previous blocks in the chain [11]. MultiChain is a platform that allows users to build and deploy private blockchain applications. It comes with a data stream feature with potential for data storage and sharing. Data streams allow users to create multiple key values, time series, or identity databases that can be used for data sharing, time stamping, and encrypted archiving [12]. Each data stream on a MultiChain blockchain consists of a list of items. These items contain information about the block, the data to be stored, and the index for the data. To store data using the streams, one needs two inputs: the data and a key as an identifier for the data.

In this paper, we focus on efficient storage and querying of genomic data access logs using blockchain technology. One of the biggest caveats of blockchain technology is the inefficiency of storing and querying data due to the potential for chains to reach large sizes. The required storage space and computational power of blockchains is greater than a centralized database application due to the blocks needing to contain information of the rest of the chain before them. The decentralized system also creates a higher latency during storage and retrieval of data. Moreover, blockchains with “proof-of-stakes” or “zero-knowledge” technologies require cryptographic verification of all transactions, which makes them extremely slow [13]. Therefore, it is challenging to use this technology for answering real-life problems. Here, we used blockchain technology for storing and querying genomic data access logs by taking advantage of the data stream feature of MultiChain. We developed a heuristic trick, in which we swapped the information that goes into the key and the data fields, making our solution efficient in both insertion and query of the data. Specifically, we stored the genomic dataset usage logs in the key field of a stream item, while indexing this data in the data field. We used minimal indexing to allow for rapid storage of the data. For querying information on the blockchain, we created data frames from the chain instances that allow for efficient querying within the instance. We also provide an alternative solution to overcome the scalability problem of the linearly increasing memory with the increasing amount of data. In this alternative solution, we indexed the data with the transaction ids and queried based on these indices.

## Methods

In this study, we aimed to design a time/space efficient data structure and mechanisms to store and retrieve the logs based on a blockchain by using the MultiChain-1.0.4 platform. The logs were stored on a small blockchain network consisting of four nodes. Each line in the data access log needed to be saved individually as one transaction and all log data and intermediate data had to be saved on-chain. No off-chain data storage was allowed.

### Blockchain in a nutshell

As described earlier, a blockchain is a collection of blocks that can be analogous to an append-only list. To ensure the immutability of the blockchain, every time a transaction is added to the system a group of miners needs to validate it. Blocks hold batches of valid transactions. A transaction moves a piece of value from a wallet address to a new wallet address. A wallet address is an alphanumeric identifier for a possible destination on the chain. Unique addresses are used for transactions; however, a user can have multiple addresses. The transaction is considered to be valid and added to a block when every party in the network (i.e., miners) validates that the sender has a sufficient amount of value and the address of the sender and the initiater of the transaction are the same. The addition of the block to the chain generates a transaction ID using a cryptographic hash function. This ID depends on various features of the chain such as the content of the block, time, and previous transaction IDs; therefore, mining blocks (i.e., approval of transaction IDs) requires time and resources. To add complexity to the transaction hashes, some blockchain platforms utilize concepts like proof-of-work. The process of proof-of-work is to search for a piece of data that is extremely difficult to produce, which is called “nonce” (i.e., number only used once). Nonce becomes the part of the transaction ID that the miners look for, and thus increases the difficulty of the mining process.

### Data streams in multiChain

MultiChain data streams allow a blockchain to be used as a general purpose database. Blockchain provides time stamping, notarization, and immutability to the stored data. A MultiChain blockchain can contain any number of streams. The data published in every stream is stored by every node in the network. Each data stream on a MultiChain blockchain consists of a list of items. Each item in the stream contains the following information, as a JSON object [12]:

- A publisher (string)
- A key (between 1-256 ASCII character, excluding whitespace and single/double quotes) (string)
- Data (hex string)
- A transaction ID (string)
- Blocktime (integer)
- Confirmations (integer)

**Fig. 1:**
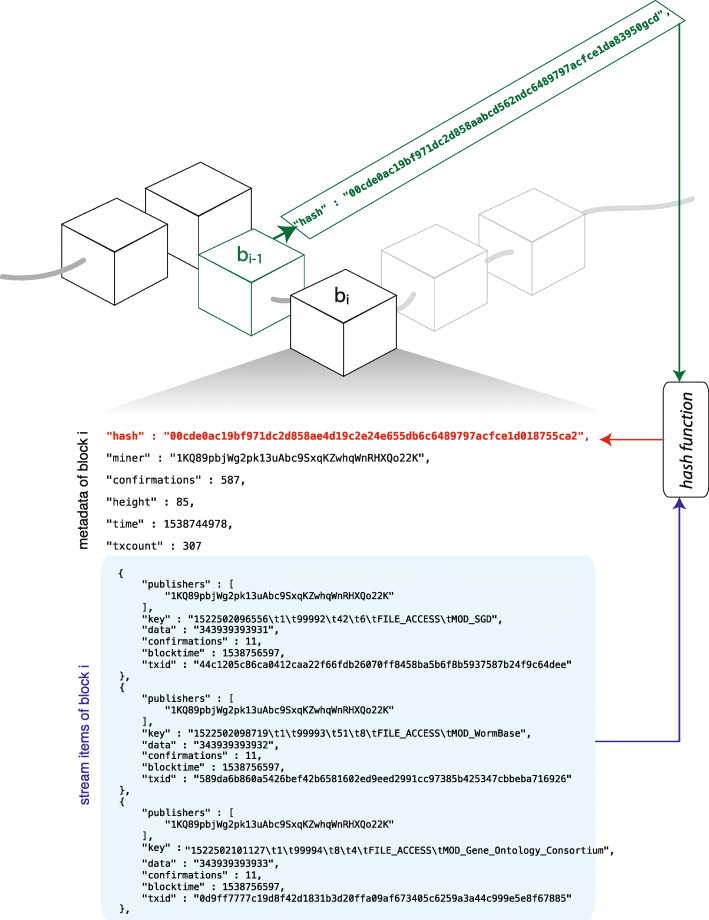
Blockchain and data streams. A blockchain is represented as the collection of blocks. Each block contains information about the transactions, which contain the data streams and the hash of the previous blocks

When a server connects to an existing chain through the MultiChain platform, it is assigned a wallet address for that chain. Multichain addresses differ from Bitcoin addresses, such that addresses created on one MultiChain blockchain cannot be valid on a second chain. This prevents an accidental operation from being performed on one chain that was intended for another [12]. When a data stream is created on a chain, servers with a wallet address for the chain may be granted permission to subscribe and publish to the data stream. Their wallet address for the chain is their publisher ID on the stream. To publish an item to a stream, the publisher must provide a key in the form of a string, and some data in the form of a hex as an input. An example stream item is shown in Fig. 1. Another property of MultiChain data streams is that requirement of miners can be turned off. This is because the stream items are added to a block based on the time the item is published [12].

#### Chain creation

**Fig. 2:**
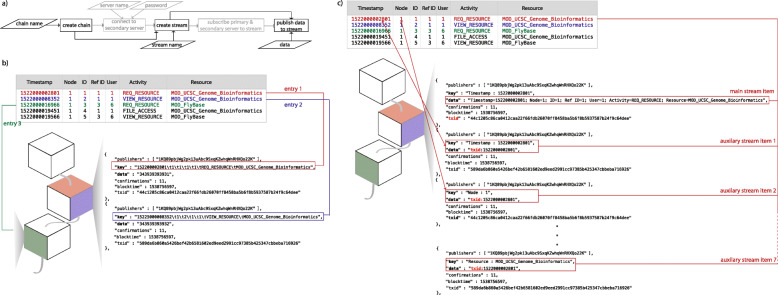
Data insertion to data streams. **a** Flowchart of the data insertion using multiple nodes in the server. **b** Example of how entries in the log files are represented in data streams in the challenge solution. Entires are appended to the blocks based on the timestamp, therefore, for example, some entries will be in block i, while other go into block j. **c** Example of how entries in the log files are represented in data streams in the bigmem solution


This step is common to both solutions we propose. Users on a primary node (server) provide a chain name and a stream name, and optionally, a second node address for which they have secure-shell (SSH) credentials. If no secondary server is supplied, the application calls MultiChain commands on the primary node to create a chain, initialize the chain, create a stream, and then subscribe the node to the stream. If a secondary server address is supplied, the application creates and initializes the chain, and then prompts the user for their password for SSH access to the second server and requests access to connect to the chain. The application then grants access to the second server from the first server. From there, it provides SSH access into the second server, and connects to the chain. On the first server, a stream is created and the first and second servers are subscribed to the stream. A flowchart of this process and the representation of the log file in the data stream are depicted in Fig. 2.

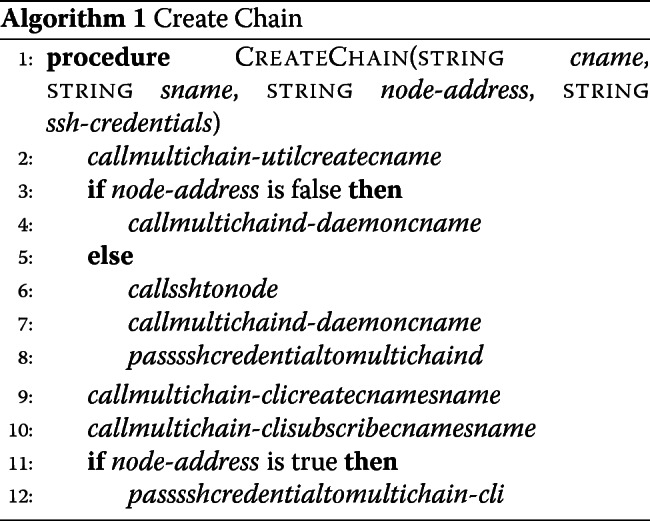


#### Challenge solution: storing data in data streams

We stored the genomic access logs as the keys of the stream items of a block. This can be done due to the short string lengths of each line in the log files, as a key can be a maximum of 256 characters in length. However, we note that if data that is larger than 256 characters needs to be stored, this solution can still be effective by using string compression techniques with a time and memory overhead. For querying, we first used the MultiChain API call **liststreamitems** to retrieve all of the stream items from a blockchain. We then parsed the returned JSON object to grab the keys from the stream items. The novelty of this solution is that we stored the data in the key field of the stream item, instead of the data field. As data field stores the data in a hex format, converting the pulled hex into plain text takes another operation and increases the query time. After parsing all of the keys in the stream, we stored the data locally in a Python Pandas data frame for further querying, after which we discarded the data frame (see Additional file 1).

**Insertion** MultiChain data streams allow for keys of up to 256 characters in length, excluding white space and single/double quotes. Each row of the genomic access logs is no more than 94 characters in length, so our solution was to first convert each tab-separated row into a single string, using the literal characters, and then publish each row to the stream as its own key, followed by the row number as the data-hex. The algorithm for inserting usage logs from text files into an existing MultiChain data stream consists of the following steps: (1) convert each row in the file to be compatible with the MultiChain key feature and (2) publish each row as a separate transaction to the key of a data stream on a chain.


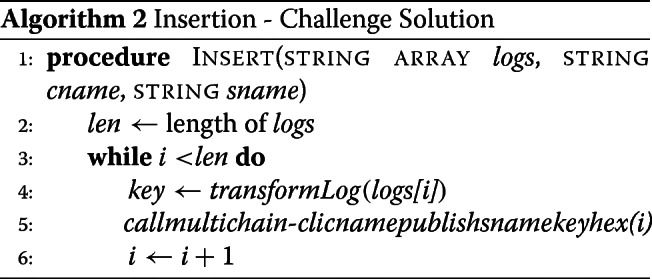


#### Challenge solution: querying data from data streams

**Fig. 3:**
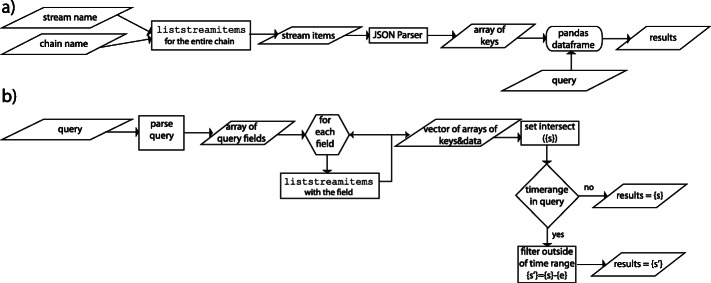
Querying. **a** Flowchart of the data query process in the challenge solution. **b** Flowchart of the data query process in the bigmem solution


The querying options in the MultiChain-1.0.4 API are limited. Stream items could be queried on transaction IDs, timestamps, or stream keys. Additionally, while the software allows for querying on streams using keys, it could not query partial keys or keys containing wildcards. Our solution, therefore, was to list all stream items, grab the keys, and create a data frame of all of the keys, which could then be queried on. The algorithm for querying usage logs from a data stream on a MultiChain consists of the following steps, as shown in the flowchart in Fig. 3. We first download the stream items locally to the memory. This creates a JSON object per stream items. After parsing the keys, we create a data frame from the stream item keys. We then perform queries on the data frame by creating a dictionary from the user query. If the user requests sorted results, then the results are returned after sorting them (see Additional file 1).

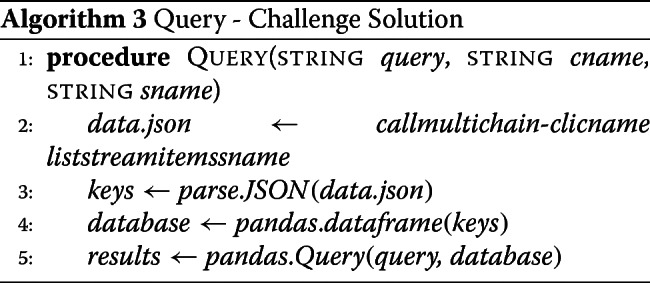


#### Alternate bigmem solution: storing data in data streams

In this solution, instead of keeping the data in the key field of the streams, we used the data-hex field to keep the data, while using the timestamps as the key.

**Insertion** In our insertion process, we store a main and several auxillary records per log entry. We first store the entire entry to the stream as the main record and get a unique transaction id. For each field in a entry, we insert an auxiliary record, using field:value as key, and timestamp:txid of main record as value (hex encoded). For example, if our record contained user 6, we would store an auxilary record mapping user:6 to the transaction id of the main record. We do this for all columns of the data, in this case 7. This increases the insertion time and storage, but comes in handy when we do the queries with less memory requirement.


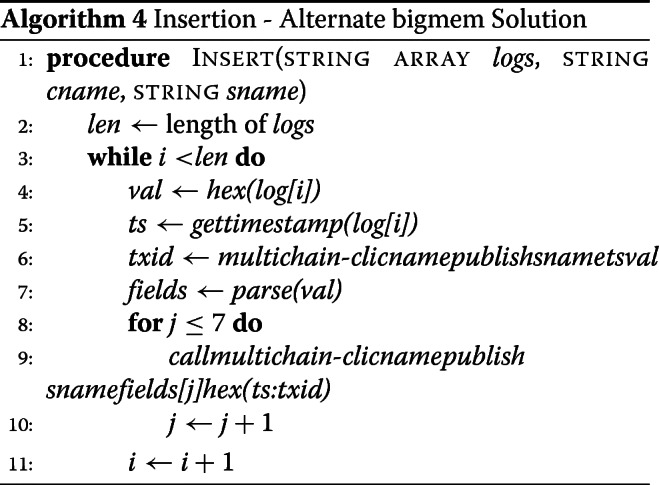


#### Alternate bigmem solution: querying data from data streams

In this solution, we take advantage of indexing we created with the transaction ids. For each query element, we first find all auxiliary records that match, and save their values as a set for each element. We then take the set intersection. This will give us timestamp:txid of all main records that matched all the query elements, apart from the start and end time criteria (if any). We filter the resulting set by start and end time, if necessary. For each surviving element of the filtered set, we extract the transaction ids from timestamp:txid, and query for main record. The set of records returned is our query result.

## Results

The limitations of blockchain-based data storage methods are time and space efficiency and the scalability of the solution. Here, we present two proof-of-concept solutions for genomic access logs up to 1,000,000 entries. Although our challenge solution scales linearly for most of the variables we evaluated, the required storage and memory cost may not scale up for solving real-life problems with billions of entries. Therefore, we provided a memory efficient alternative solution that can be used when the log sizes reaches large numbers at the expense of run time overhead.

### Accuracy

**Fig. 4:**
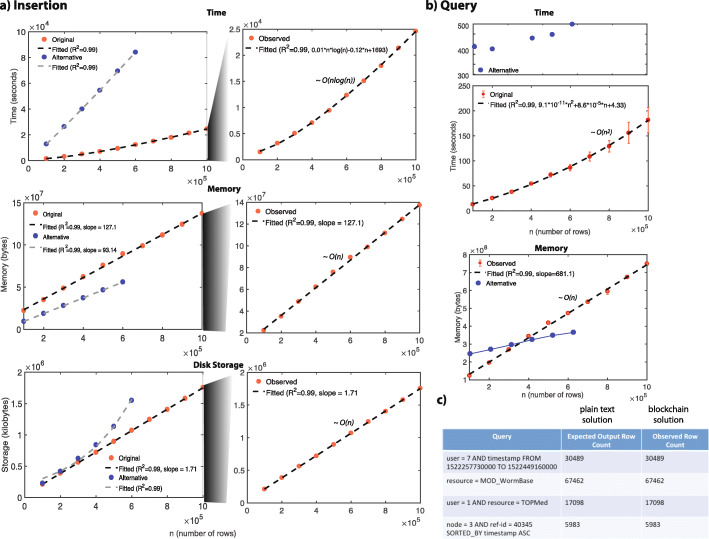
Results. **a** Time, memory and storage complexity of data storage in a blockchain. **b** Time and memory complexity of querying. The curve is fitted using the average times of querying 100 random queries and standard deviations are depicted as error bars. **c** Accuracy of example queries


We generated a random 100 queries that are a combination of different columns in the data to test the accuracy of our solution. We also tested these queries in chains that have different amount of data. We found that in a chain with increasing amounts of data, our solution showed 100% accuracy for every query in the set. Figure 4c shows that the expected number of returned rows that are calculated based on plain-text logs and using “awk” commands and the returned rows for example queries are identical for both solutions.

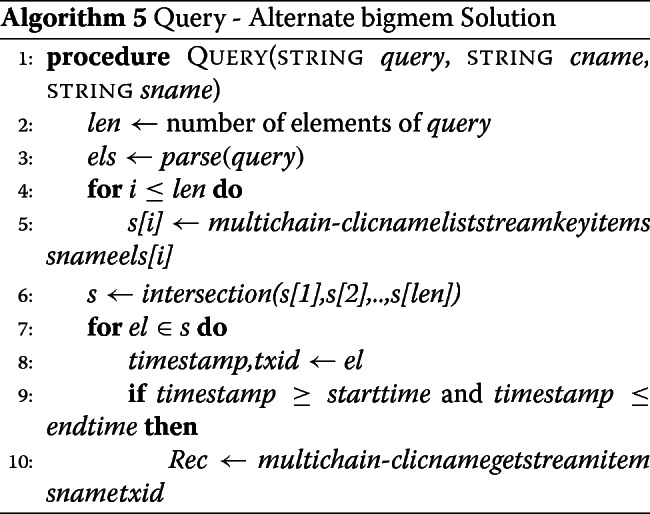


### Speed, storage and scalability

**Insertion** We measured the performance of our insertion protocol by testing (1) the time required, (2) the maximum memory required, and (3) the disk storage required to store increasing amounts of data for both solutions. We found that it takes around 24.5 minutes and 214 minutes to insert 100,000 entries of a genomic data usage log into a chain with challenge and bigmem solutions, respectively. This scales up to around 410 minutes for a log with 1,000,000 entries with an estimated complexity of *O*(*nlog*(*n*)) with the challenge solution. It scales up to 243 minutes for a log with 600,000 entries with the bigmem solution (Fig. 4a). We found that the memory requirement for inserting 100,000 genomic data usage log into a chain is around 22 MB and 9.4 MB and linearly scales up to 137 MB for a log with 1,000,000 entries with the challenge solution and scales up to 56 MB for a log with 600,000 entries with the bigmem solution (Fig. 4a). We then found that a chain with 100,000 entries takes up around 0.2 GB of space, which linearly scales up to 1.7 GB for a log with 1,000,000 entries with the challenge solution. A chain with 100,000 entries takes up around 0.3 GB of space and this requirement scales up to around 16 GB for a log with 600,000 entries with the bigmem solution (Fig. 4a).

**Querying** We measured the performance of our query protocol by testing (1) the time and (2) the maximum memory required to make a query from chains with an increasing amount of data. To measure this empirically, we used the generated 100 random queries that were a combination of different columns of the data. We calculated the running time for each query and found an average of 12.8 seconds and 140 seconds when we used a chain with 100,000 entries with the challenge and the bigmem solutions, respectively. The average time scales up to 177.5 seconds for a chain with 1,000,000 entries with the challenge solution, while it scales up to 500 seconds for a chain with 600,000 entries with the bigmem solution (Fig. 4b). We calculated that the memory requirement for querying from a chain with 100,000 entries is around 123 MB linearly scales up to 749 MB for data with 1,000,000 entries with the challenge solution. On the other hand, while the memory requirement of the bigmem solution for a chain with 100,000 entries is still larger than those with challenge solution (251 MB), it scales only up to 359 MB for a chain with 600,000 entries (Fig. 4b).

**Fig. 5:**
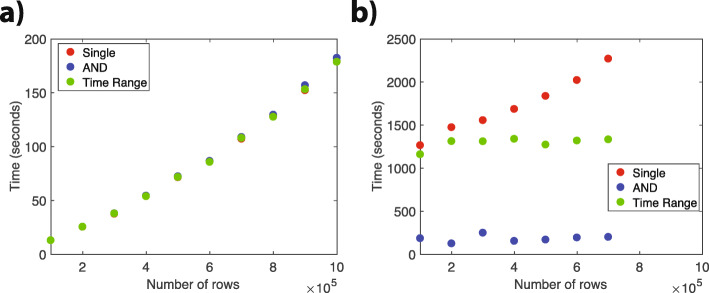
Results. **a** Comparison of different query times in the challenge solution. **b** Comparison of different query times in the bigmem solution

**Overhead from queries with “AND” and “time ranges”:** We also investigated whether the required time for a query is based on the type of query for both solutions (Fig. 5). For that, we divided our randomly generated queries in three categories: (1) single: queries with a single field, (2) AND: queries with multiple fields, (3) queries with time ranges. We found that our challenge solution uses similar amount of times to do the queries in these three categories, while alternative solution requires different amount of time to query for these three categories. This is largely because of the overhead from pulling the data from the blockchain. If the query is performed after pulling the data from blockchain in the memory locally, then the query takes a smaller amount of time. If the query requires multiple operations within the MultiChain API, then it adds an overhead to the required time.

## Discussion

Auditing access to sensitive genomic data is essential to protect private information from potential malicious activities. Such auditing is made possible by careful logging and mining of user access; hence, the security and integrity of the logs are essential. The iDASH Secure Genome Analysis Competition in 2018 proposed a challenge to develop blockchain-based ledgering solutions to log and query the user activities of accessing genomic datasets across multiple sites [14]. Such a solution would protect data from the consequences of a single point of failure by providing a decentralized infrastructure. This challenge also showcased the underlying technology of blockchain for applications in genomics beyond its reputation as being a cryptocurrency.

Decentralization is the delegation of an authority from a central entity such as a bank to a larger group. Decentralized storage is the storage of data in multiple nodes. Increasing attention has been focused on decentralized data storage options after recent data leaks at companies such as Equifax and Facebook Cambridge Analytica. Current blockchain applications provide numerous options for decentralized data storage. The simplest option is to store data in blockchain itself, which MultiChain data streams also offer. However, storing data in the streams is different than traditional blockchain-based data storage, where data is embedded in the raw transactions. Another feature of MultiChain API that is different from other applications is that miners are not necessary. That is, if a user is creating a private blockchain they can store data rapidly without needing to wait for mining. This allows MultiChain data streams to be much faster than other applications. Overall, the biggest advantage of storing the data in a blockchain, whether in a data stream or through validated transactions, is the immutability of the system, which provides robustness. However, in some cases immutability could be a disadvantage for data storage. This is because data is stored in the chain forever and can be seen by anyone in the network. This also creates problems regarding storage size because no data can be deleted; thus, the blockchain will grow rapidly and require publicly available hard drive space.

In this study, we focused on the security aspect of data storage by taking advantage of the time stamping, notarization, and immutability aspects of blockchain. However, the stored data can be seen by anyone in the network. This creates a privacy bottleneck, particularly if the data to be stored contains sensitive information such as genomic variants of a patient or electronic health records. MultiChain also provides stream confidentiality, which allows users to selectively reveal data on a blockchain when sensitive information is stored. This is solved by encrypting the data before storing and timestamping the chain. The key for the encrypted data can be available to a subset of the users in the network. This is done by using a combination of symmetric and asymmetric cryptographic techniques and making use of being able to create multiple streams for public keys, private data, and public data. This property allows users to create different access groups by creating multiple streams.

Although storing data in MultiChain data streams is a convenient solution, the computation on the data such as querying is not well developed yet. The first version of MultiChain (iDASH requirement) does not have a module that can search for multiple keys in the data stream. This creates inefficiency when the data is searched for multiple fields. To overcome this problem, we stored the data in the key field of the stream items. This allowed us to list all of the keys in the stream and store the list locally as a data frame. Our challenge solution works excellent for the question posed in the challenge, such that the data need to be stored is shorter than 256 characters so that it can be stored as key and the total amount of data can be locally kept in the memory for efficient querying. Such approach cuts from the query time tremendously. However, one might imagine that holding the keys of the entire chain becomes memory inefficient when large datasets are stored. We addressed this problem by creating a solution with indexing the data with transaction IDs. We found that, although the required memory still increases with increasing amount of data, the rate of increase is much smaller than our challenge solution and scales to smaller memory requirement when large sizes of data is stored with an overhead to the query time and chain size.

In sum, we demonstrated two proof-of-concept applications in solving problems related to storing genomic access logs (also see Additional file 1). We emphasize that our solution is not limited to genomic access logs, and other biomedical data such as electronic health records or genomic variant files can be stored using the concepts in this study. Indeed, several early projects are using blockchain technology for genomics applications; however, their success remains to be seen [10]. How blockchain technology can solve problems in genomics and biomedical informatics and how it relates to scientific computing, distributed data systems, and privacy and security will likely be important questions as the technology develops.

## Conclusions

In conclusion, we showed that genomic access log files can be stored and queried efficiently using a blockchain platform.

## Supplementary information

**Additional file 1** Supplementary information. A pdf file including four additional figures describing how MultiChain blockchain work and how we insert and query data from blockchain.

## Abbreviations

iDASH: Integrating data for analysis, anonymization, and sharing
bigmem: Big memory
SSH: Secure shell
API: Application programming interface
JSON: JavaScriptoObject notation
