# Supplementary material for: Using blockchain to log genome dataset access: efficient storage and query
Source: BMC Med Genomics. 2020 Jul 21;13(Suppl 7):78. doi: 10.1186/s12920-020-0716-z (PMC7372787; doi:10.1186/s12920-020-0716-z)
Supplement: Supplementary file 1 — Additional file 1 Supplementary information. A pdf file including four additional figures describing how MultiChain blockchain work and how we insert and query data from blockchain. [file 12920_2020_716_MOESM1_ESM.pdf]

Supplementary Information

List of Figures

1 MultiChain blockchain explained 1
2 Challenge solution 2
3 Bigmem solution 3
4 Example query for bigmem solution 4

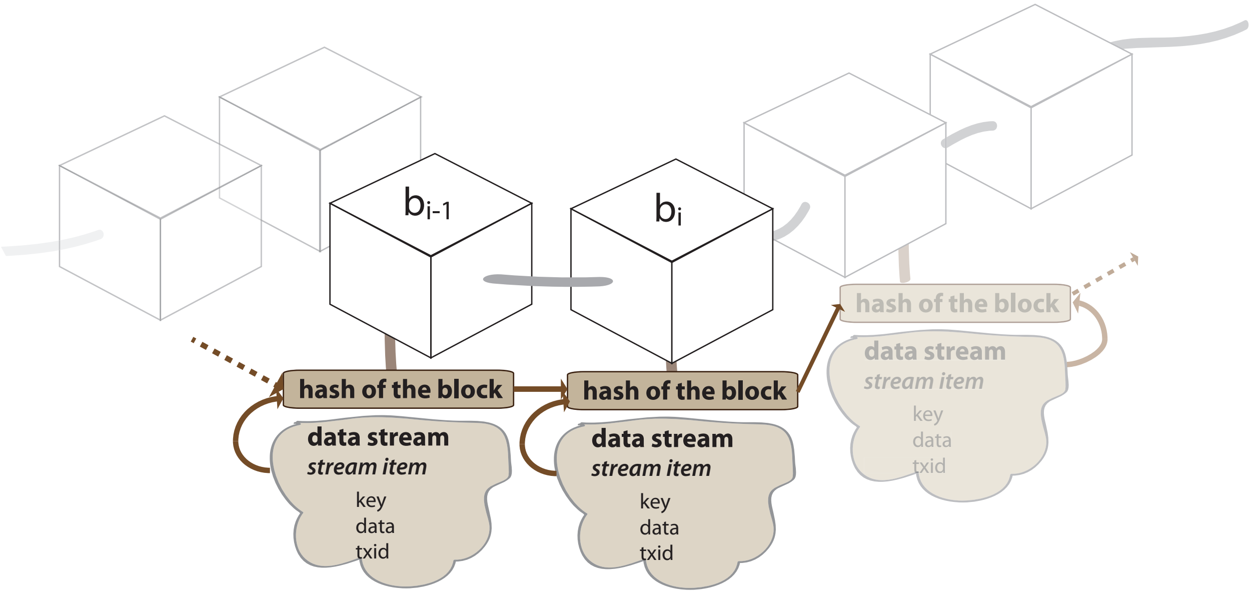

Supplementary Figure 1: MultiChain is a platform to allow users to create private blockchains. These chains come with the property of data streams, where users can push data as an append-only list. Each stream item allow a single key:value item that the user can query from. Here we depict that the data and the transactions in the previous blocks and the data and the transactions in the current block are encoded in the hash of the current block.

	Timestamp	Node	ID	Ref ID	User	Activity	Resource
entry 1	1522000002801	1	1	1	1	REQ_RESOURCE	MOD_UCSC_Genome_Bioinformatics
entry 2	1522000008352	1	2	1	1	VIEW_RESOURCE	MOD_UCSC_Genome_Bioinformatics
entry 3	1522000016966	1	3	3	6	REQ_RESOURCE	MOD_FlyBase
entry 4	1522000019451	1	4	1	1	FILE_ACCESS	MOD_UCSC_Genome_Bioinformatics
						⋮	

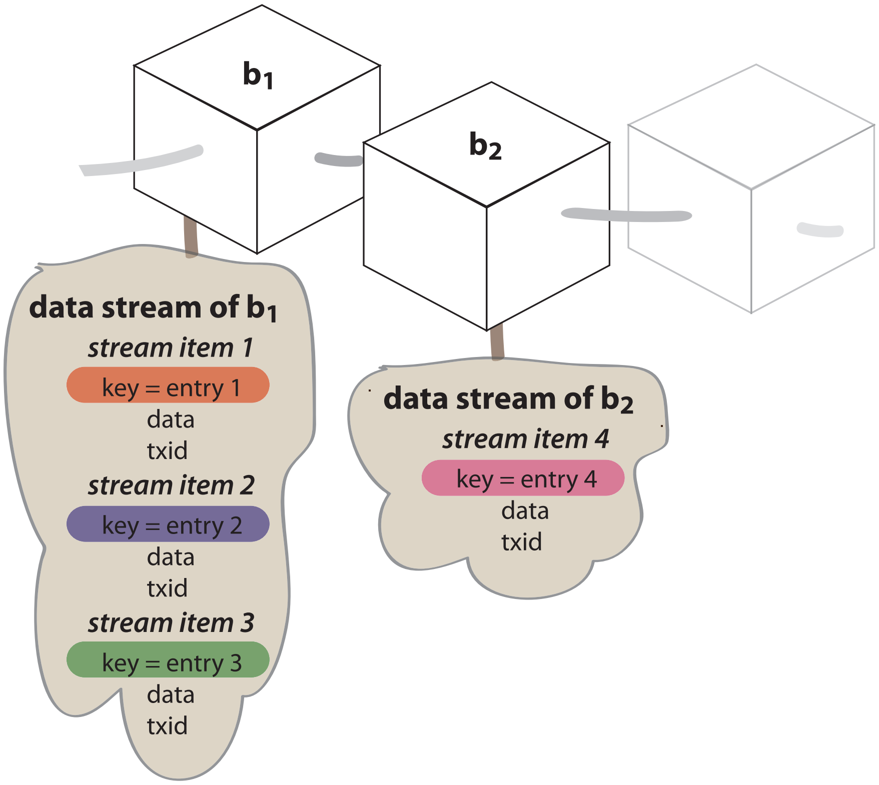

Supplementary Figure 2: Depiction of the solution that was used for the 2018 iDASH Secure Genome Analysis Challenge. In this solution, we inserted each entry of a log file as a key to a stream item. Stream items are appended to a block based on the timestap that the data is pushed to the chain, hence each block might have a different numbers of stream item stored. We query the stream items from the chain and download all of the keys to the local memory. We then create a dataframe from these keys and locally query the dataframe. This creates a large memory overhead when the size of the data is large.

	Timestamp	Node	ID	Ref ID	User	Activity	Resource
entry 1	1522000002801	1	1	1	1	REQ_RESOURCE	MOD_UCSC_Genome_Bioinformatics
entry 2	1522000008352	1	2	1	1	VIEW_RESOURCE	MOD_UCSC_Genome_Bioinformatics
						⋮	

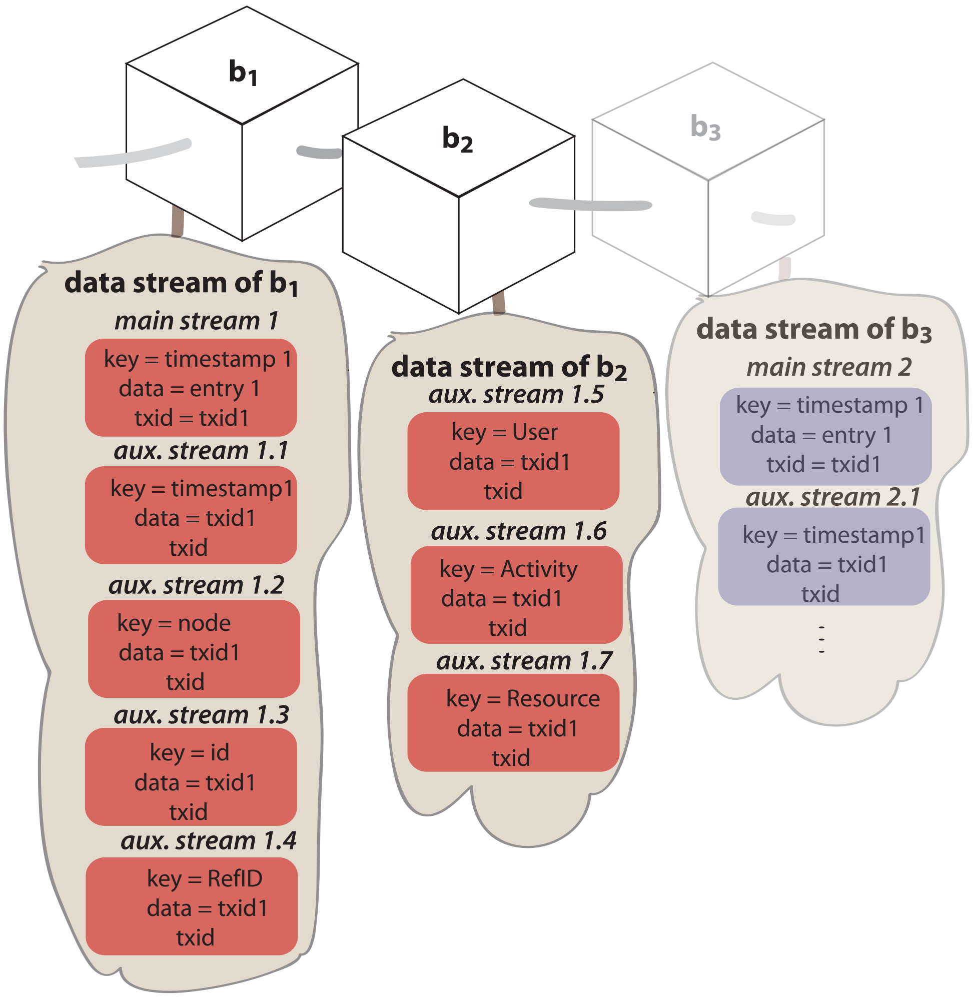

Supplementary Figure 3: Depiction of an alternative solution to remedy in creasing memory overhead for chains with large amount of data. In this solution, we first encode record and insert as main record into stream, using timestamp as key (not used), and get a unique transaction id. For each field in record (7 total), we insert an auxiliary record, using field:value as key, and timestamp:txid of main record as value (hex encoded). For query, for each query element in the query, we find all auxiliary records that match, and save their values as a set for each element. We then take the set intersection. This will give us timestamp:txid of any main records that matched all the query elements. We filter the resulting set s for auxiliary records that pass the start and end time criteria. For each surviving element of s, we extract the txid from timestamp:txid, and query for the main record. the set of records returned is our query result.

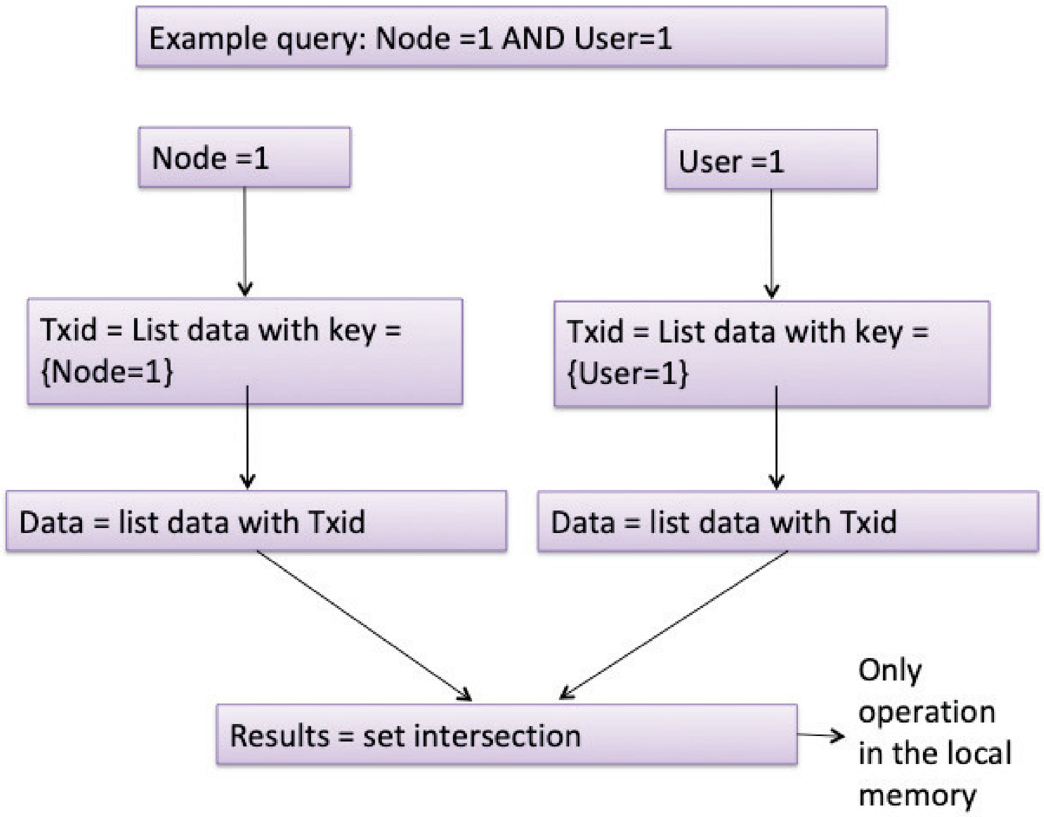

Supplementary Figure 4: Example query and how it is executed based on the algorithm described in 3.
